# Cross-Reactivity as a Mechanism Linking Infections to Stroke

**DOI:** 10.3389/fneur.2019.00469

**Published:** 2019-05-14

**Authors:** Guglielmo Lucchese, Agnes Flöel, Benjamin Stahl

**Affiliations:** ^1^Department of Neurology, University of Greifswald, Greifswald, Germany; ^2^Department of Computing, Goldsmiths, University of London, London, United Kingdom; ^3^Department of Neurology, Charité Universitätsmedizin Berlin, Berlin, Germany; ^4^Department of Neurophysics, Max Planck Institute for Human Cognitive and Brain Sciences, Leipzig, Germany; ^5^Psychologische Hochschule Berlin, Berlin, Germany

**Keywords:** stroke, infections, cross-reactivity, peptides, inflammation

## Abstract

The relevance of infections as risk factor for cerebrovascular disease is being increasingly recognized. Nonetheless, the pathogenic link between the two entities remains poorly understood. Consistent with recent advances in medicine, the present work addresses the hypothesis that infection-induced immune responses may affect human proteins associated with stroke. Applying established procedures in bioinformatics, the pathogen antigens and the human proteins were searched for common sequences using pentapeptides as probes. The resulting data demonstrate massive peptide sharing between infectious pathogens—such as *Chlamydia pneumoniae, Streptococcus pneumoniae, Tannerella forsythia, Haemophilus influenzae*, Influenza A virus, and Cytomegalovirus—and human proteins related to risk of ischemic and hemorrhagic stroke. Moreover, the shared peptides are also evident in a number of epitopes experimentally proven immunopositive in the human host. The present findings suggest cross-reactivity as a potential mechanistic link between infections and stroke.

## Introduction

When considered separately from other cardiovascular diseases, stroke ranks fifth among all causes of death (1) and, critically, its incidence is on the rise (2).

The etiology of stroke is multifactorial with various environmental and genetic risk factors. Hypertension, diabetes and insulin resistance, smoking, dyslipidemia, obesity, heavy alcohol consumption, atrial fibrillation, and carotid stenosis are all established and well-investigated modifiable risk factors of stroke (3–5).

Additionally, there is evidence that environmental factors may also increase risk of stroke, including viral and bacterial infections, such as periodontitis (6) and respiratory infections (7), and infection with *Chlamydia pneumoniae* (8) or Cytomegalovirus (9). However, relatively little is known so far about the role of different pathogens as well as the molecular basis and the mechanisms that potentially link infections to stroke.

Here we set out to investigate whether or not infections can induce immune responses capable of cross-reacting with human proteins that, when altered, have been associated with stroke. Our hypothesis was that immune responses induced by infectious agents might cross-react with crucial stroke-related proteins, thus contributing to the multifactorial pathogenesis of cerebrovascular disease.

To address this hypothesis, we analyzed pathogens, as well as proteins that are known to be associated with increased risk of ischemic and hemorrhagic stroke by searching for common peptides that might underlie cross-reactions.

Specifically, we analyzed antigens from the following pathogens that have been reported to have a possible influence on stroke: the periodontal bacterium *Tannerella forsythia* (10), *Haemophilus influenza* (11), *Streptococcus pneumoniae* (7), *Chlamydia pneumoniae* (8), Influenza A viruses (12, 13), and Human Cytomegalovirus (9).

## Methods

We analyzed the amino acid (aa) primary sequence of pathogen antigens (with short name and UniProt ID in parentheses):

- Surface antigen repeat/outer membrane protein (OMP; UniProtKB: A0A0F7WYE8_CHLPN) from *Chlamydia pneumoniae*;
- Pneumococcal vaccine antigen A (PVAA;UniProtKB: PVAA_STRR6) from *Streptococcus pneumoniae*;
- Surface antigen BspA (BspA; UniProtKB: O68831_TANFO) from *Tannerella forsythia*;
- Outer membrane antigenic lipoprotein B (LPPB; UniProtKB: LPPB_HAEIN) from *Haemophilus influenzae* (strain ATCC 51907);
- Hemagglutinin (HA H1N1; UniProtKB: HEMA_I34A1) from Influenza A virus (strain A/Puerto Rico/8/1934 H1N1);
- Hemagglutinin (HA H5N1; UniProtKB: HEMA_I96A0) from Influenza A virus (strain A/Goose/Guangdong/1/1996 H5N1);
- Hemagglutinin (HA H3N2; UniProtKB: HEMA_I68A6) from Influenza A virus (strain A/Northern Territory/60/1968 H3N2); and
- 65 kDa phosphoprotein (pp65; UniProtKB: PP65_HCMVM) from Human Cytomegalovirus (HCMV; strain Merlin).

The primary sequence of pathogen antigens was dissected into partially overlapping pentapeptides with a one-residue-offset: i.e., MFKRI, FKRIR, KRIRR, and so on. Then, each pentapeptide was analyzed for occurrences within a library consisting of primary sequences of human proteins involved in stroke. The human protein library was *a priori* chosen from the UniProtKB Database (https://www.uniprot.org) (14) using the keyword “stroke.” We obtained an unbiased list of 74 human proteins (in)directly associated with stroke (Table S1). Stroke-related proteins are indicated as UniProtKB entry names throughout the present article, except when discussed in detail. The pathogen antigens and the human proteins were searched for common sequences using the pentapeptide as a probe unit because a pentapeptide is an immunobiological determinant sufficient for epitope-paratope interaction and for inducing specific immune responses (15–18).

The immunologic potential of the shared peptides was analyzed using the Immune Epitope Database (IEDB; www.iedb.org) (19). All evaluations were based only on epitopic sequences that had been experimentally validated as immunopositive in the human host.

This linear peptide similarity analysis procedure has been used and described before (20, 21).

## Results

In a detailed overview, Table 1 shows that 49 out of the 74 human stroke-related proteins share peptide sequences with antigens from pathogens that proved to be (in)directly involved in stroke (6–10). It can be seen that

- The pathogen vs. human peptide overlap is unexpectedly high when considering that the probability for two proteins to share a pentapeptide is 1 out of 20^−5^, that is, 0.0000003125 or close to zero.
- The peptide overlap varies widely, with *T. forsythia* BspA and Influenza A HA H3N2 being the pathogen more and less involved in the peptide sharing, respectively.
- The high number of stroke-related proteins involved in the viral peptide overlap precludes a detailed protein-by-protein analysis. However, an example worth noting is the human ATP-binding cassette sub-family C member nine (ABCC9 or SUR2) that shares peptide sequences with all of the pathogen antigens analyzed, with the exception of the Influenza A HA H3N2 virus. ABCC9 is a subunit of ATP-sensitive potassium channels (K_ATP_) that can form cardiac and smooth muscle-type KATP channels with KCNJ11 and mediates neuroprotection (22).

**Table 1:** Peptide sharing between pathogen antigens and human proteins that have been associated with stroke^1^.

**Shared peptides^a^^,^^b^**	**Human protein involved in the peptide ^Sharing^^b^^,^^c^**
***C. pneumoniae*** **OMP:**	
ITNYL	**ABCC9**. ATP-binding cassette sub-family C member 9
**RKFLL**	CCM2. Cerebral cavernous malformations 2 protein
**RKFLL**; **KGFVS**	CCM2L. Cerebral cavernous malformations 2 protein-like
ASSVD; LEHNQ	CSF1R. Macrophage colony-stimulating factor 1 receptor
IALHL	**DAPK1**. Death-associated protein kinase 1
SEGKT	FA5. Coagulation factor V
PTTGI	GNAQ. Guanine nucleotide-binding protein G(q) subunit alpha
EGPCG	HTRA1. Serine protease HTRA1
NTTAE	KCNE2. Potassium voltage-gated channel subfamily E member 2
GFRCL; LRSSA	NOTC3. Neurogenic locus notch homolog protein 3
VSAAG	**NU155**. Nuclear pore complex protein Nup155
SGLGG	PAWR. PRKC apoptosis WT1 regulator protein
SGNQV	PDE4D. cAMP-specific 3′,5′-cyclic phosphodiesterase 4D
GYFAS	**RN213**. E3 ubiquitin-protein ligase RNF213
DSPRT; SPRTP	SAMH1. Deoxynucleoside triphosphate triphosphohydrolase SAMHD1
***S. pneumoniae*** **PVAA:**	
LAMIY	**ABCC9**. ATP-binding cassette sub-family C member 9
TVAPL; VAPLL	KCNA5. Potassium voltage-gated channel subfamily A member 5
AQNGK	KLOT. Klotho
SASGS	LMNA. Prelamin-A/C
LVLAV	NMDE2. Glutamate receptor ionotropic, NMDA 2B
IQTLT	NU5M. NADH-ubiquinone oxidoreductase chain 5
***T. forsythia*** **BspA:**	
AWTAR; SGTKT	A4. Amyloid-beta A4 protein
GLTTI; LTITN	**ABCC9**. ATP-binding cassette sub-family C member 9
TLSAL	BI1. Bax inhibitor 1
APGRA	CO4A2. Collagen alpha-2(IV) chain
GKKAV	COQ8A. Atypical kinase COQ8A, mitochondrial
IIFVS	CXA5. Gap junction alpha-5 protein
NCGAL	GATA5. Transcription factor GATA-5
HSLQS	GATA6. Transcription factor GATA-6
LGATA; GATAQ	IL4. Interleukin-4
DALTT	ITIH4. Inter-alpha-trypsin inhibitor heavy chain H4
AGGAL; VTTIG	KCNQ1. Potassium voltage-gated channel subfamily KQT member 1
TAPDA	KRIT1. Krev interaction trapped protein 1
EGFAL	LYAM3. P-selectin
VTQNP	NMDE2. Glutamate receptor ionotropic, NMDA 2B
DGVNT; SGTTG	NOTC3. Neurogenic locus notch homolog protein 3
GLFLL	SCN4B. Sodium channel subunit beta-4
TLPNS	SCN5A. Sodium channel protein type 5 subunit alpha
TLPDG; VTLPN	SYLM. Probable leucine—tRNA ligase, mitochondrial
LPDAL; LTLSA; SGLTS; TLPDA	**ZFHX3**. Zinc finger homeobox protein 3
***H. influenzae*** **LPPB:**	
TSNFP; GIDIS	ABCC9. ATP-binding cassette sub-family C member 9
LLLPL	ACE. Angiotensin-converting enzyme
SFLLL; TTTVS	ANF. Natriuretic peptides A
AQPAF	CSF1R. Macrophage colony-stimulating factor 1 receptor
ILVAD	ENPP4. Bis(5′-adenosyl)-triphosphatase ENPP4
**VTSSV**	GATA6. Transcription factor GATA-6
GNLII	ITIH4. Inter-alpha-trypsin inhibitor heavy chain H4
PGANG; SGSRG	KCNA5. Potassium voltage-gated channel subfamily A member 5
APDYS; PDYSK; DYSKI; TYTPG	KRIT1. Krev interaction trapped protein 1
SNVGG; SPSVP	NU155. Nuclear pore complex protein Nup155
**AYLAG**	PDE3A. cGMP-inhibited 3′,5′-cyclic phosphodiesterase A
LLPLS;**AYLAG**; **VTSSV**; QEVKA	RN213. E3 ubiquitin-protein ligase RNF213
GPIKS	SCN5A. Sodium channel protein type 5 subunit alpha
KKSFL	SYLM. Probable leucine–tRNA ligase, mitochondrial
**Influenza A HA H1N1:**	
DGVKL	ADA2. Adenosine deaminase 2
LLVSL	ATP6. ATP synthase subunit a
ENAYV	**ABCC9**. ATP-binding cassette sub-family C member 9
ASSLV	PDE3A. cGMP-inhibited 3′,5′-cyclic phosphodiesterase A
**AELLV**; **ELLVL**; LLVLL; LVLLV	**DAPK1**. Death-associated protein kinase 1
TVLEK; YVSVV; QTPLG; FLDIW	**RN213**. E3 ubiquitin-protein ligase RNF213
TSNAS	NMDE2. Glutamate receptor ionotropic, NMDA 2B
CALAA	GAS6. Growth arrest-specific protein 6
LLVLL;YAADQ; KVDGV	KLOT. Klotho
EELRE	LMNA. Prelamin-A/C
YSEES	**ZFHX3**. Zinc finger homeobox protein 3
**Influenza A HA H5N1:**	
LLAIV	AL5AP. Arachidonate 5-lipoxygenase-activating protein
**LLLAI**	**ABCC9**. ATP-binding cassette sub-family C member 9
AQDIL; ISGVK	PDE4D. cAMP-specific 3′,5′-cyclic phosphodiesterase 4D
**LLLAI**	CYTC. Cystatin-C
QRLVP; **AELLV**; **ELLVL**	**DAPK1**. Death-associated protein kinase 1
ILEKT; LKHLL; **VSSAC**	**RN213**. E3 ubiquitin-protein ligase RNF213
EGGWQ	KLOT. Klotho
**SLALA**	NU5M. NADH-ubiquinone oxidoreductase chain 5
KIVLL; LVLAT	**NU155**. Nuclear pore complex protein Nup155
ARLNR; SIYST	KCNQ1. Potassium voltage-gated channel subfamily KQT member 1
**VSSAC**	SCN1B. Sodium channel subunit beta-1
VPEWS	TBX5. T-box transcription factor TBX5
SVAGW	S19A2. Thiamine transporter 1
**SLALA**	GATA5. Transcription factor GATA-5
**Influenza A HA H3N2:**	
GGSNA; **AELLV**	**DAPK1**. Death-associated protein kinase 1
INSNG	SAMH1. Deoxynucleoside triphosphate triphosphohydrolase SAMHD1
KITYG	MYL4. Myosin light chain 4
LLGDP	KCNA5. Potassium voltage-gated channel subfamily A member 5
ISFAI	HTRA1. Serine protease HTRA1
VLNVT	SCN3B. Sodium channel subunit beta-3

1 *References in Table S1 (Supplementary Material)*.

a *Viral/bacterial antigens are described under Methods. Further details at https://www.uniprot.org (14)*.

b *Multiple occurrences in bold*.

c *Human proteins given as UniProt entry and name. Further details at https://www.uniprot.org (14)*.

In summary, Table 1 describes a peptide platform that connects the infectious agents under analysis human proteins related to stroke.

Subsequently, in order to define the immunologic potential of the shared peptides, we conducted analyses throughout the peptide immunome cataloged in the Immune Epitope Database (IEDB; www.iedb.org) (19). The search was finalized to identify epitopic sequences corresponding to (or containing) the peptide sequences shared between stroke-related infectious agents and stroke-related human proteins. It was found that a great number of the shared peptides listed in Table 1 are also distributed through hundreds of epitopic sequences with an immunological potential. A list of such epitopic sequences is reported in Table 2.

**Table 2:** Epitopes immunopositive in the human host and containing peptide sequences common to antigens from stroke-related pathogens and human proteins associated with stroke.

**1**	**2**	**1**	**2**	**1**	**2**	**1**	**2**
58129	SGLTSlf	459109	sLLPLShlv	554097	lvesyTLPDGrii	634993	mplhVAPLLaa
66225	tSGLTSl	466105	gtdpLVLAV	554098	lvesyTLPDGriikv	637221	slsdLLVSL
66817	ttSGLTS	467909	LLLPLnlev	554195	mfehlINSNGikpv	638032	tpLLLPLaa
69631	vLLVSLgai	471133	SPRTPppltv	555093	qgDGVKLhlkakaev	638320	vhEGPCGisy
79809	ELLVLLenertld	471667	tlAELLVLL	555672	rlsrlqekEELRE	642301	eqklnrypASSLVvvr
113324	dgFLDIWtynAELLV	475091	aekeNTTAEl	557456	vlvesyTLPDGrii	642391	etpvyLGATAgmrll
113533	idlwsynAELLVal	476155	ASSLVdtpk	559587	gnENAYVkngklhls	644577	iekdlilLGATAvedrl
143137	tSASGSgedaidesg	476488	avdDGVNTf	563134	evAELLVkh	644669	iifgASSVDf
150977	eeaklnreeiDGVKL	478710	GLTTIlknv	570114	AELLVshga	645002	inhvvSVAGWgisd
151075	ynAELLVLLenertl	482488	neILVADty	571918	EELRElaesw	645986	kpTLSALpsplvtsg
151076	ypgdfidyEELREql	482780	nrypASSLV	573110	haCALAAslw	648442	qgkdlqqYVSVVvld
163409	setdaLRSSA	482921	pkyvksnrLVLATg	574223	kpagpLGATA	649953	sldrSLALAaeep
164690	fLLVLLdyqgmlp	483957	reSNVGGiqql	575691	nrypASSLVvv	691126	klnrypASSLVvvr
164772	gvdtGIDIShsdf	485150	rySGNQVlf	577376	rVLNVTnlef	691145	kpTLSALpsplvts
178609	avvvlkrLPDALadg	485418	sedpsGKKAVl	577393	rvqGYFASf	691655	neqklnrypASSLVv
182409	stvASSLVLLVSLga	487836	TVLEKfrylpk	578014	SPRTPnrsv	692295	skpTLSALpsplvtsg
182414	yqilaiystvASSLV	488193	vifftSGTTGfpk	578015	SPRTPsntp	694354	eskrKKSFLlcig
184585	lrTTTVSgkl	488539	vVAPLLrkv	578016	SPRTPsntpsa	695746	pltspttsqLRSSAp
190442	hELLVLvkkaq	488926	ypASSLVvv	578044	SPSVPktsa	695843	pSASGSsgntptppn
194133	llaAWTARa	489284	LLLPLpvpa	579718	vyLKHLLpk	695894	pttsqLRSSApshaq
196781	sILEKTsay	490154	araLLLPLl	581635	aeLLLPLkvl	696039	qLRSSApshaqtpwp
213534	kssSGTKTtk	492412	irfgLLPLSm	581817	anatLLPLSl	699244	aqhSGLGGvshy
223189	ldLLLPLnl	492430	irfSGTTGqm	582446	eeLLLPLly	699571	asiVAPLLl
223510	pelLLLPL	492987	lepcsrlLLLPLl	583971	iamhaaLLLAI	699594	aslVAPLLi
223880	seeLLLPLl	493322	mpepasrclLLLPL	584143	ildksVAPLL	699595	aslVAPLLl
226701	yvftLLVSL	493610	plLLVSLw	584764	kgVSAAGilek	700497	deVTLPNvv
240338	sASSLVnldslv	493947	qrqqLLVSL	584945	klrEELREk	700729	difqQTPLGr
243935	FLDIWtyna	495137	srlLLLPLl	587249	prclLLVLL	700885	dpSGTKTcidtkeg
419699	gpprlLLLPL	495478	traLLLPLl	587956	raqggLLVLL	702273	EELREkyrry
423950	alLKHLLsy	496437	eirTVLEKl	589061	rrgELLVLr	702274	EELRElanky
424543	fLLPLSlif	497010	tpvyLGATAgmrll	590120	srsKIVLLv	703553	erySGNQVl
426499	nfkAWTARy	504703	clyvLLLAI	590169	stsSPSVPk	704661	evvtSEGKTk
430136	fspdLLGDPdny	505049	elitILEKT	590856	vpglcllvll	705071	fAQPAFml
430260	fTVLEKfry	505187	fasVAPLLef	590986	vsgTLPDGhmp	706516	fsLLPLShl
435575	rrlLLLPLl	505268	flaptfSGLTSi	592071	dttyinhvvSVAGWg	710491	ieILEKTttiy
435576	rrlLLLPLll	507273	mpllLLLPL	594553	atiEELRErvw	711114	ipingSPRTPr
436423	apfGPIKSidm	507346	mtlLLLPL	595684	gLLAIVkv	711645	itnvAELLV
437494	gAGGALfvhrd	507484	palSPSVPl	600696	qttTVLEKy	712552	kfpeiVAPLL
437792	gSGLGGltdk	510341	ypASSLVv	601366	rTLPDGthel	713254	klkikelre
440682	SPRTPvspvkf	511858	ardSLALArpkssdvy	601983	sryLLPLSalgtvag	713313	kLLLAIktk
441210	TVLEKvyel	518869	inhvvSVAGWgisdg	602564	tiEELRErvw	716976	ltneKIVLL
442404	apaAPGRAl	519272	itILEKTvspdrle	610338	QEVKAitkl	720288	qhyelcSGNQV
443560	erySGNQVlf	520276	kqqeLLVSL	611939	rrvkAELLV	721762	rgaLLPLSi
444320	glsLLPLSek	521984	lrqaAGGALqvvhsr	614291	asfapISFAIk	722338	rlLLLPLl
445722	kpkhPTTGI	523925	qaAGGALqvvhsrql	615815	evAELLVrh	722416	rlprLLVLL
448068	rvrvPTTGI	532960	mPTTGIney	616957	glvnSGLTSv	723746	rvpaLLVLL
448661	SPRTPpqrf	535612	ELLVLrqkhsepsrf	620774	kvleAELLVLr	723778	rvpsLLVLL
448662	SPRTPtpfkhal	537116	nkvleAELLVLrqkh	623038	qrAGGALsi	724649	SEGKTfqly
448918	sryLLPLSa	541075	allpAGGALqh	627837	vPTTGIiey	724650	SEGKTkpli
449866	VSAAGlvqgl	541668	eEELREkqay	628836	AELLVkgyei	725019	sgdGPIKSv
453784	flplLLVLL	541699	EELREkqay	628959	ailhLLVSL	725662	slVAPLLi
456288	LLLAIiphv	544699	rggaAGGALp	630008	dvAGGALthsll	727559	TAPDAaltl
456316	LLLPLplll	545442	srvpLLLPL	632600	iLLLPLhtg	728413	TLPDGthel
457304	ntlLLVSL	548131	hLLGDPmanv	633968	lLLLPLpvpa	728445	TLSALyarr
457363	pekTLSALl	551494	esyTLPDGrii	634370	lprLLVLLa	729508	tvpdLLLPL
458091	rlAELLVsv	553367	kpvlqkyAELLV	634991	mplhVAPLL	732341	yAPDYSsrl

*Column 1: Epitopes listed according to the IEDB-ID number. Epitope details and references at www.immuneepitope.org (19). Column 2: Sequences common to pathogen antigens and human proteins related to stroke are indicated in capital letters*.

## Conclusion

Stroke risk appears to be the result of a complex combination of multiple genetic non-modifiable and environmental modifiable factors that can be further classified as either “traditional” or new, “emerging” ones (23). As highlighted by Grau et al. (24, 25), the occurrence of stroke is only partially explained by traditional modifiable cardiovascular risk factors, such as increasing blood pressure, cigarette smoking, and diabetes mellitus. Most importantly, infectious diseases appear to play a key role in contributing to the risk of stroke and are to be counted among “emerging” modifiable risk factors that receive increasing scientific interest (6–10, 23, 26, 27).

Searching for possible immunopathogenic links between infection and risk of stroke, the present study aimed to analyze the potential immunologic relationship between pathogens and human proteins that, when altered, have been associated with risk of stroke. In line with our hypothesis, we found that immune cross-reactions between infectious pathogens and human stroke-related proteins might occur, thus increasing the risk of stroke (see Tables 1, 2).

The immunologically relevant peptide sharing reported in the present study depicts a complex scenario. Some potential molecular targets of cross-reactions are proteins belonging to the cardiovascular system, thus possibly directly accounting for cerebrovascular damage. Other possible targets are proteins of the immune system, thus suggesting mechanisms resulting in immune dysregulation which could lead to cerebrovascular damage.

An example of the first type of potential targets are ion-channels, particularly potassium (K^+^) and sodium (Na^+^) channels (ABCC9, KCNE2, KCNA5, KCNQ1, SCN4B, SCN5A, SCN1B, SCN3B, see Table 1). Accordingly, a growing body of evidence points to the involvement of cardiac K^+^ and Na^+^ channel dysfunction (cardiac channelopathies as a result of genetic mutations and/or inflammatory mechanisms) in the pathogenesis of atrial fibrillation (AF), an established risk factor for stroke (28, 29). Moreover, autoantibodies targeting ion-channels may be involved in cardiac arrhythmias (30). In light of the potential cross-reactivity suggested by the observed peptide sharing, AF and subsequent stroke could result from antibodies primarily targeting epitopes of infective agents but also cross-reacting with cardiac ion channels. For instance, activating antibodies could lead to a gain-of-function of K^+^-channels and inhibiting antibodies to a loss-of-function of of Na^+^-channels. This could promote re-entry or increase susceptibility to early and/or delayed afterdepolarizations, two mechanisms that can generate AF (31, 32).

The second class of potential targets includes proteins that actively modulate the inflammatory response, such as cytokines and colony-stimulating factor receptors (IL-4, macrophage colony-stimulating factor 1 receptor, see Table 1) (33, 34). IL-4, for example, is a well-investigated tolerogenic cytokine that is able to suppress inflammatory responses and organ-specific autoimmunity in both animal models and humans (35, 36). It is then conceivable that autoantibodies downregulating the function of these proteins can promote inflammatory responses, thus increasing the risk of cerebrovascular damage and stroke. Indeed, inflammatory responses appear to be crucial in the pathogenesis of stroke by inducing atherosclerosis progression, pro-thrombotic activation, and AF—among other mechanisms (37). Inflammation can therefore be considered as one key factor underpinning the relationship between classical stroke risk factors and comorbidities. It appears that not just single infections, but overall infectious burden from multiple agents predicts stroke incidence. Moreover, poor outcome may be proportional to systemic inflammatory burden both in patients and experimental models. For instance, Influenza and Streptococcus infection seem to contribute to stroke incidence and outcome, and evidence from experimental models indicate that blocking inflammatory processes might be an effective prevention strategy (38, 39).

The increasingly recognized relevance of inflammation in stroke is consistent with a possible role of peptide sharing-based cross-reactivity as contributing factors to cerebrovascular damage. In fact, the past two decades of immunologic research have radically changed the way we think of inflammation and innate immunity. It is now known that innate immune responses can “specifically” drive the following adaptive responses through recognition of pathogen-associated molecular patterns (PAMPs) (40, 41). That is to say that peptide epitopes, cell-wall components, and other PAMPs activate immune cells already from the very first stages of immune reactions and drive inflammation. Indeed, there are examples of cross-reactivity between host and pathogen-associated molecular patterns: identical inflammasomes and toll-like receptors (TLRs) recognizing molecular fingerprints of both pathogens (the PAMPs) and injured host cells (so-called danger-associated molecular patterns; DAMPs). For instance, both bacteria LPS and HMGB1 from injured host cells activate TLR4, with consequent inflammation in various tissues including the brain (41, 42). TLR- and inflammasome-dependent pathways seem to be important drivers of inflammation, vascular disease, and reportedly contribute to stroke outcome (43, 44).

Our preliminary results underline the importance of further experimental efforts to define the molecular basis through which microbial infections might contribute to an increased risk of stroke (45–50). Future studies should evaluate immunoreactivity against the peptides shared by infectious pathogens and human stroke-related proteins in sera from stroke patients. Possibly, such serological analyses could also help identify specific markers predicting a higher risk of stroke and might therefore be useful to design preventive strategies following an infection. The ultimate translational relevance of our finding lies in the possibility of adopting effective individualized primary and secondary preventive strategies in patients at risk for stroke after infections. Generic hygienic measure, as well as antibiotic prophylaxis and vaccination campaigns have already been proposed and tested with contrasting results (37). Identifying and stratifying patients according to individual biomarker profiles would allow to personalize treatment for each patient, thus possibly increasing overall efficacy.

Until now, stroke is a leading cause of preventable death and adult disability (1–5, 47–52), but preventive strategies mostly concentrate on traditional cardiovascular risk factors (53–56). Moreover, “cryptogenic” stroke (i.e., ischemic stroke with no obvious cause) poses a challenge in terms of primary and secondary prevention (57, 58).

Given the burden of cerebrovascular disease, and the potential to identify immunological markers that may then serve as prognostic indicators of risk of cerebrovascular damage after an infection, our results justify further intensive research on the cross-reactive link between infections and risk of stroke.

## Author Contributions

GL formulated the hypothesis, analyzed the data and wrote the manuscript. GL, AF, and BS interpreted the data, revised and finalized the manuscript.

### Conflict of Interest Statement

AF received consulting fees from Bayer and Novartis and honoraria for oral presentations from Novartis, Böhringer-Ingelheim, Lilly, and Biogen Idec (unrelated to current research). The remaining authors declare that the research was conducted in the absence of any commercial or financial relationships that could be construed as a potential conflict of interest.
