# Supplementary material for: Cross-Reactivity as a Mechanism Linking Infections to Stroke
Source: Front Neurol. 2019 May 14;10:469. doi: 10.3389/fneur.2019.00469 (PMC6528689; doi:10.3389/fneur.2019.00469)
Supplement: Supplementary file 1 [file Table_1.DOCX]

**Table S1.** Peptide sharing between pathogen antigens and human proteins that have been associated with stroke (with references).

| **Shared Peptides^1,2^** | **Human Protein Involved in the Peptide ^Sharing2,3^** | **Refs.** |
| --- | --- | --- |
| ***C. pneumoniae* OMP:** | | |
| ITNYL | **ABCC9**. ATP-binding cassette sub-family C member 9 | [1, 2] |
| **RKFLL** | CCM2. Cerebral cavernous malformations 2 protein | [3] |
| **RKFLL**; KGFVS | CCM2L. Cerebral cavernous malformations 2 protein-like | [3] |
| ASSVD; LEHNQ | CSF1R. Macrophage colony-stimulating factor 1 receptor | [4] |
| IALHL | **DAPK1**. Death-associated protein kinase 1 | [5] |
| SEGKT | FA5. Coagulation factor V | [6] |
| PTTGI | GNAQ. Guanine nucleotide-binding protein G(q) subunit alpha | [7] |
| EGPCG | HTRA1. Serine protease HTRA1 | [8] |
| NTTAE | KCNE2. Potassium voltage-gated channel subfamily E member 2 | [9] |
| GFRCL; LRSSA | NOTC3. Neurogenic locus notch homolog protein 3 | [10] |
| VSAAG | **NU155**. Nuclear pore complex protein Nup155 | [11] |
| SGLGG | PAWR. PRKC apoptosis WT1 regulator protein | [12, 13] |
| SGNQV | PDE4D. cAMP-specific 3',5'-cyclic phosphodiesterase 4D | [14] |
| GYFAS | **RN213**. E3 ubiquitin-protein ligase RNF213 | [15] |
| DSPRT; SPRTP | SAMH1. Deoxynucleoside triphosphate triphosphohydrolase SAMHD1 | [16] |
| ***S. pneumoniae* PVAA** | | |
| LAMIY | **ABCC9.** ATP-binding cassette sub-family C member 9 | [1, 2] |
| TVAPL; VAPLL | KCNA5. Potassium voltage-gated channel subfamily A member 5 | [17] |
| AQNGK | KLOT. Klotho | [18] |
| SASGS | LMNA. Prelamin-A/C | [19] |
| LVLAV | NMDE2. Glutamate receptor ionotropic, NMDA 2B | [20, 21] |
| IQTLT | NU5M. NADH-ubiquinone oxidoreductase chain 5 | [22] |
| ***T.* *forsythia* BspA:** | | |
| AWTAR; SGTKT | A4. Amyloid-beta A4 protein | [23] |
| GLTTI; LTITN | **ABCC9**. ATP-binding cassette sub-family C member 9 | [1, 2] |
| TLSAL | BI1. Bax inhibitor 1 | [24] |
| APGRA | CO4A2. Collagen alpha-2(IV) chain | [25] |
| GKKAV | COQ8A. Atypical kinase COQ8A, mitochondrial | [26] |
| IIFVS | CXA5. Gap junction alpha-5 protein | [27] |
| NCGAL | GATA5. Transcription factor GATA-5 | [28] |
| HSLQS | GATA6. Transcription factor GATA-6 | [29] |
| LGATA; GATAQ | IL4. Interleukin-4 | [30] |
| DALTT | ITIH4. Inter-alpha-trypsin inhibitor heavy chain H4 | [31] |
| AGGAL; VTTIG | KCNQ1. Potassium voltage-gated channel subfamily KQT member 1 | [32] |
| TAPDA | KRIT1. Krev interaction trapped protein 1 | [33] |
| EGFAL | LYAM3. P-selectin | [30] |
| VTQNP | NMDE2. Glutamate receptor ionotropic, NMDA 2B | [20, 21] |
| DGVNT; SGTTG | NOTC3. Neurogenic locus notch homolog protein 3 | [10] |
| GLFLL | SCN4B. Sodium channel subunit beta-4 | [34] |
| TLPNS | SCN5A. Sodium channel protein type 5 subunit alpha | [35] |
| TLPDG; VTLPN | SYLM. Probable leucine--tRNA ligase, mitochondrial | [36] |
| LPDAL; LTLSA; SGLTS; TLPDA | **ZFHX3**. Zinc finger homeobox protein 3 | [37] |
| ***H. influenzae* LPPB:** | | |
| TSNFP; GIDIS | ABCC9. ATP-binding cassette sub-family C member 9 | [1, 2] |
| LLLPL | ACE. Angiotensin-converting enzyme | [38] |
| SFLLL; TTTVS | ANF. Natriuretic peptides A | [39] |
| AQPAF | CSF1R. Macrophage colony-stimulating factor 1 receptor | [4, 40] |
| ILVAD | ENPP4. Bis(5'-adenosyl)-triphosphatase ENPP4 | [41] |
| **VTSSV** | GATA6. Transcription factor GATA-6 | [29] |
| GNLII | ITIH4. Inter-alpha-trypsin inhibitor heavy chain H4 | [30] |
| PGANG; SGSRG | KCNA5. Potassium voltage-gated channel subfamily A member 5 | [17] |
| APDYS; PDYSK; DYSKI; TYTPG | KRIT1. Krev interaction trapped protein 1 | [33] |
| SNVGG; SPSVP | NU155. Nuclear pore complex protein Nup155 | [11] |
| **AYLAG** | PDE3A. cGMP-inhibited 3',5'-cyclic phosphodiesterase A | [42] |
| LLPLS; **AYLAG**; **VTSSV**; QEVKA | RN213. E3 ubiquitin-protein ligase RNF213 | [15] |
| GPIKS | SCN5A. Sodium channel protein type 5 subunit alpha | [35] |
| KKSFL | SYLM. Probable leucine--tRNA ligase, mitochondrial | [36] |
| **Influenza A HA H1N1:** | | |
| DGVKL | ADA2. Adenosine deaminase 2 | [43] |
| LLVSL | ATP6. ATP synthase subunit a | [44] |
| ENAYV | **ABCC9.** ATP-binding cassette sub-family C member 9 | [1, 2] |
| ASSLV | PDE3A. cGMP-inhibited 3',5'-cyclic phosphodiesterase A | [38] |
| **AELLV**; **ELLVL**; LLVLL; LVLLV | **DAPK1.** Death-associated protein kinase 1 | [5] |
| TVLEK; YVSVV; QTPLG; FLDIW | **RN213.** E3 ubiquitin-protein ligase RNF213 | [15] |
| TSNAS | NMDE2. Glutamate receptor ionotropic, NMDA 2B | [20, 21] |
| CALAA | GAS6. Growth arrest-specific protein 6 | [45] |
| LLVLL;YAADQ; KVDGV | KLOT. Klotho | [18] |
| EELRE | LMNA. Prelamin-A/C | [19] |
| YSEES | **ZFHX3.** Zinc finger homeobox protein 3 | [37] |
| **Influenza A HA H5N1:** | | |
| LLAIV | AL5AP. Arachidonate 5-lipoxygenase-activating protein | [46] |
| **LLLAI** | **ABCC9.** ATP-binding cassette sub-family C member 9 | [1, 2] |
| AQDIL; ISGVK | PDE4D. cAMP-specific 3',5'-cyclic phosphodiesterase 4D | [14] |
| **LLLAI** | CYTC. Cystatin-C | [47] |
| QRLVP; **AELLV**; **ELLVL** | **DAPK1.** Death-associated protein kinase 1 | [5] |
| ILEKT; LKHLL; **VSSAC** | **RN213.** E3 ubiquitin-protein ligase RNF213 | [15] |
| EGGWQ | KLOT. Klotho | [18] |
| **SLALA** | NU5M. NADH-ubiquinone oxidoreductase chain 5 | [22] |
| KIVLL; LVLAT | **NU155.** Nuclear pore complex protein Nup155 | [11] |
| ARLNR; SIYST | KCNQ1. Potassium voltage-gated channel subfamily KQT member 1 | [32] |
| **VSSAC** | SCN1B. Sodium channel subunit beta-1 | [48] |
| VPEWS | TBX5. T-box transcription factor TBX5 | [49] |
| SVAGW | S19A2. Thiamine transporter 1 | [50, 51] |
| **SLALA** | GATA5. Transcription factor GATA-5 | [28] |
| **Influenza A HA H3N2:** | | |
| GGSNA; **AELLV** | **DAPK1.**Death-associated protein kinase 1 | [5] |
| INSNG | SAMH1. Deoxynucleoside triphosphate triphosphohydrolase SAMHD1 | [16] |
| KITYG | MYL4. Myosin light chain 4 | [52] |
| LLGDP | KCNA5. Potassium voltage-gated channel subfamily A member 5 | [17] |
| ISFAI | HTRA1. Serine protease HTRA1 | [8] |
| VLNVT | SCN3B. Sodium channel subunit beta-3 | [53] |

^1^ Viral/bacterial antigens are described under Methods. Further details at https://www.uniprot.org [54].

^2^ Multiple occurrences in bold

^3^ Human proteins given as UniProt entry and name. Further details at https://www.uniprot.org [54].

1. Liu, R., et al., *Cerebrovascular Safety of Sulfonylureas: The Role of KATP Channels in Neuroprotection and the Risk of Stroke in Patients With Type 2 Diabetes.* Diabetes, 2016. **65**(9): p. 2795-809.

2. Stoller, D.A., et al., *Cardiomyocyte sulfonylurea receptor 2-KATP channel mediates cardioprotection and ST segment elevation.* Am J Physiol Heart Circ Physiol, 2010. **299**(4): p. H1100-8.

3. Rosen, J.N., et al., *ccm2-like is required for cardiovascular development as a novel component of the Heg-CCM pathway.* Dev Biol, 2013. **376**(1): p. 74-85.

4. Jin, W.N., et al., *Depletion of microglia exacerbates postischemic inflammation and brain injury.* J Cereb Blood Flow Metab, 2017. **37**(6): p. 2224-2236.

5. Wang, S., et al., *DAPK1 Signaling Pathways in Stroke: from Mechanisms to Therapies.* Mol Neurobiol, 2017. **54**(6): p. 4716-4722.

6. Casas, J.P., et al., *Meta-analysis of genetic studies in ischemic stroke: thirty-two genes involving approximately 18,000 cases and 58,000 controls.* Arch Neurol, 2004. **61**(11): p. 1652-61.

7. Pinto, A., M. Sahin, and P.L. Pearl, *Epileptogenesis in neurocutaneous disorders with focus in Sturge Weber syndrome.* F1000Res, 2016. **5**.

8. Verdura, E., et al., *Heterozygous HTRA1 mutations are associated with autosomal dominant cerebral small vessel disease.* Brain, 2015. **138**(Pt 8): p. 2347-58.

9. Yang, Y., et al., *Identification of a KCNE2 gain-of-function mutation in patients with familial atrial fibrillation.* Am J Hum Genet, 2004. **75**(5): p. 899-905.

10. Joutel, A., et al., *Notch3 mutations in CADASIL, a hereditary adult-onset condition causing stroke and dementia.* Nature, 1996. **383**(6602): p. 707-10.

11. Zhang, X., et al., *Mutation in nuclear pore component NUP155 leads to atrial fibrillation and early sudden cardiac death.* Cell, 2008. **135**(6): p. 1017-27.

12. Lekic, T., et al., *Protease-activated receptor 1 and 4 signal inhibition reduces preterm neonatal hemorrhagic brain injury.* Stroke, 2015. **46**(6): p. 1710-3.

13. Culmsee, C., et al., *Evidence for the involvement of Par-4 in ischemic neuron cell death.* J Cereb Blood Flow Metab, 2001. **21**(4): p. 334-43.

14. Jorgensen, C., et al., *Phosphodiesterase4D (PDE4D)--A risk factor for atrial fibrillation and stroke?* J Neurol Sci, 2015. **359**(1-2): p. 266-74.

15. Liu, W., et al., *Identification of RNF213 as a susceptibility gene for moyamoya disease and its possible role in vascular development.* PLoS One, 2011. **6**(7): p. e22542.

16. Thiele, H., et al., *Cerebral arterial stenoses and stroke: novel features of Aicardi-Goutieres syndrome caused by the Arg164X mutation in SAMHD1 are associated with altered cytokine expression.* Hum Mutat, 2010. **31**(11): p. E1836-50.

17. Olson, T.M., et al., *Kv1.5 channelopathy due to KCNA5 loss-of-function mutation causes human atrial fibrillation.* Hum Mol Genet, 2006. **15**(14): p. 2185-91.

18. Arking, D.E., et al., *Association between a functional variant of the KLOTHO gene and high-density lipoprotein cholesterol, blood pressure, stroke, and longevity.* Circ Res, 2005. **96**(4): p. 412-8.

19. Gonzalez-Quereda, L., et al., *LMNA mutation in progeroid syndrome in association with strokes.* Eur J Med Genet, 2011. **54**(6): p. e576-9.

20. Tu, W., et al., *DAPK1 interaction with NMDA receptor NR2B subunits mediates brain damage in stroke.* Cell, 2010. **140**(2): p. 222-34.

21. Stanca, D.M., et al., *GFAP and antibodies against NMDA receptor subunit NR2 as biomarkers for acute cerebrovascular diseases.* J Cell Mol Med, 2015. **19**(9): p. 2253-61.

22. Liolitsa, D., et al., *Is the mitochondrial complex I ND5 gene a hot-spot for MELAS causing mutations?* Ann Neurol, 2003. **53**(1): p. 128-32.

23. Obici, L., et al., *A novel AbetaPP mutation exclusively associated with cerebral amyloid angiopathy.* Ann Neurol, 2005. **58**(4): p. 639-44.

24. Krajewska, M., et al., *Endoplasmic reticulum protein BI-1 modulates unfolded protein response signaling and protects against stroke and traumatic brain injury.* Brain Res, 2011. **1370**: p. 227-37.

25. Jeanne, M., et al., *COL4A2 mutations impair COL4A1 and COL4A2 secretion and cause hemorrhagic stroke.* Am J Hum Genet, 2012. **90**(1): p. 91-101.

26. Mollet, J., et al., *CABC1 gene mutations cause ubiquinone deficiency with cerebellar ataxia and seizures.* Am J Hum Genet, 2008. **82**(3): p. 623-30.

27. Yang, Y.Q., et al., *Novel connexin40 missense mutations in patients with familial atrial fibrillation.* Europace, 2010. **12**(10): p. 1421-7.

28. Yang, Y.Q., et al., *Mutational spectrum of the GATA5 gene associated with familial atrial fibrillation.* Int J Cardiol, 2012. **157**(2): p. 305-7.

29. Yang, Y.Q., et al., *GATA6 loss-of-function mutation in atrial fibrillation.* Eur J Med Genet, 2012. **55**(10): p. 520-6.

30. Zee, R.Y., et al., *Polymorphism in the P-selectin and interleukin-4 genes as determinants of stroke: a population-based, prospective genetic analysis.* Hum Mol Genet, 2004. **13**(4): p. 389-96.

31. Kashyap, R.S., et al., *Inter-alpha-trypsin inhibitor heavy chain 4 is a novel marker of acute ischemic stroke.* Clin Chim Acta, 2009. **402**(1-2): p. 160-3.

32. Chen, Y.H., et al., *KCNQ1 gain-of-function mutation in familial atrial fibrillation.* Science, 2003. **299**(5604): p. 251-4.

33. Kehrer-Sawatzki, H., et al., *Mutation and expression analysis of the KRIT1 gene associated with cerebral cavernous malformations (CCM1).* Acta Neuropathol, 2002. **104**(3): p. 231-40.

34. Li, R.G., et al., *Mutations of the SCN4B-encoded sodium channel beta4 subunit in familial atrial fibrillation.* Int J Mol Med, 2013. **32**(1): p. 144-50.

35. Darbar, D., et al., *Cardiac sodium channel (SCN5A) variants associated with atrial fibrillation.* Circulation, 2008. **117**(15): p. 1927-35.

36. Li, R. and M.X. Guan, *Human mitochondrial leucyl-tRNA synthetase corrects mitochondrial dysfunctions due to the tRNALeu(UUR) A3243G mutation, associated with mitochondrial encephalomyopathy, lactic acidosis, and stroke-like symptoms and diabetes.* Mol Cell Biol, 2010. **30**(9): p. 2147-54.

37. Zaw, K.T.T., et al., *Association of ZFHX3 gene variation with atrial fibrillation, cerebral infarction, and lung thromboembolism: An autopsy study.* J Cardiol, 2017. **70**(2): p. 180-184.

38. Bennion, D.M., et al., *Serum activity of angiotensin converting enzyme 2 is decreased in patients with acute ischemic stroke.* J Renin Angiotensin Aldosterone Syst, 2016. **17**(3).

39. Hodgson-Zingman, D.M., et al., *Atrial natriuretic peptide frameshift mutation in familial atrial fibrillation.* N Engl J Med, 2008. **359**(2): p. 158-65.

40. Battisti, C., et al., *Hereditary diffuse leukoencephalopathy with axonal spheroids: three patients with stroke-like presentation carrying new mutations in the CSF1R gene.* J Neurol, 2014. **261**(4): p. 768-72.

41. Albright, R.A., et al., *Molecular basis of purinergic signal metabolism by ectonucleotide pyrophosphatase/phosphodiesterases 4 and 1 and implications in stroke.* J Biol Chem, 2014. **289**(6): p. 3294-306.

42. Maass, P.G., et al., *PDE3A mutations cause autosomal dominant hypertension with brachydactyly.* Nat Genet, 2015. **47**(6): p. 647-53.

43. Zhou, Q., et al., *Early-onset stroke and vasculopathy associated with mutations in ADA2.* N Engl J Med, 2014. **370**(10): p. 911-20.

44. Burrage, L.C., et al., *Mitochondrial myopathy, lactic acidosis, and sideroblastic anemia (MLASA) plus associated with a novel de novo mutation (m.8969G>A) in the mitochondrial encoded ATP6 gene.* Mol Genet Metab, 2014. **113**(3): p. 207-12.

45. Munoz, X., et al., *Human vitamin K-dependent GAS6: gene structure, allelic variation, and association with stroke.* Hum Mutat, 2004. **23**(5): p. 506-12.

46. Helgadottir, A., et al., *The gene encoding 5-lipoxygenase activating protein confers risk of myocardial infarction and stroke.* Nat Genet, 2004. **36**(3): p. 233-9.

47. Levy, E., M. Jaskolski, and A. Grubb, *The role of cystatin C in cerebral amyloid angiopathy and stroke: cell biology and animal models.* Brain Pathol, 2006. **16**(1): p. 60-70.

48. Watanabe, H., et al., *Mutations in sodium channel beta1- and beta2-subunits associated with atrial fibrillation.* Circ Arrhythm Electrophysiol, 2009. **2**(3): p. 268-75.

49. Wang, Z.C., et al., *Prevalence and Spectrum of TBX5 Mutation in Patients with Lone Atrial Fibrillation.* Int J Med Sci, 2016. **13**(1): p. 60-7.

50. Shaw-Smith, C., et al., *Recessive SLC19A2 mutations are a cause of neonatal diabetes mellitus in thiamine-responsive megaloblastic anaemia.* Pediatr Diabetes, 2012. **13**(4): p. 314-21.

51. Karimzadeh, P., T. Moosavian, and H. Moosavian, *Recurrent Stroke in a Child with TRMA Syndrome and SLC19A2 Gene Mutation.* Iran J Child Neurol, 2018. **12**(1): p. 84-88.

52. Orr, N., et al., *A mutation in the atrial-specific myosin light chain gene (MYL4) causes familial atrial fibrillation.* Nat Commun, 2016. **7**: p. 11303.

53. Wang, P., et al., *Functional dominant-negative mutation of sodium channel subunit gene SCN3B associated with atrial fibrillation in a Chinese GeneID population.* Biochem Biophys Res Commun, 2010. **398**(1): p. 98-104.

54. UniProt Consortium, T., *UniProt: the universal protein knowledgebase.* Nucleic Acids Res, 2018. **46**(5): p. 2699.
